# The Non‐Invasive Diagnostic Modality for the Detection of Oral Squamous Cell Carcinoma by an Infrared Sensor

**DOI:** 10.1002/cnr2.70436

**Published:** 2026-03-05

**Authors:** Shahrzad Rahimizadeh Nahavandi, Arghavan Tonkaboni, Soheila Manifar, Mohammad Shirkhoda, Amir Parham Pirhadi Rad

**Affiliations:** ^1^ Department of Oral Health Sciences, Faculty of Dentistry The University of British Columbia Vancouver Canada; ^2^ Department of Oral and Maxillofacial Medicine, School of Dentistry Tehran University of Medical Sciences Tehran Iran; ^3^ Grupo de investigación en patología Oral médico quirúrgica University of Santiago de Compostela Spain; ^4^ Department of Surgery, Cancer Institute of Iran Tehran University of Medical Sciences Tehran Iran; ^5^ School of Biomedical Engineering University of British Columbia Vancouver Canada; ^6^ Department of Pathology & Laboratory Medicine, Faculty of Medicine University of British Columbia Vancouver Canada

**Keywords:** early detection of cancer, head and neck cancer, oral cancer, thermography

## Abstract

**Objectives:**

Oral squamous cell carcinoma (OSCC) is the most common cancer in the oral and maxillofacial region. While the 5‐year survival rate ranges from 75% to 94% when detected early, the majority of cases are diagnosed at an advanced stage, where survival drops to 20%–40%, underscoring the critical need for improved early detection strategies. This study aimed to non‐invasively detect OSCC by measuring the thermal difference between carcinogenic tissue and healthy mucosa using an infrared sensor and to assess the accuracy of this diagnostic modality.

**Materials and Methods:**

A novel intraoral infrared device was designed and manufactured to non‐invasively measure intraoral tissue temperature. Twenty participants (13 males and 7 females) were examined, including 10 patients and 10 healthy individuals. The temperature of the lesion and contralateral healthy mucosa in the patients' group as well as both sides of the tongue in the control group were measured. The temperature differences were analyzed using the *t*‐test. The accuracy of the device was evaluated using the receiver operating characteristic (ROC) curve.

**Results:**

A significant difference was observed in the temperature of the tumoral tissue and healthy mucosa in the patients' group (*p* < 0.001). The assessment of the device's accuracy in detecting OSCC revealed that a temperature differential greater than 0.97°C between the measured sides indicates the potential presence of a lesion on the higher temperature side (sensitivity = 1, specificity = 1). Regions exhibiting temperatures higher than 38.42°C were identified as potentially indicating the presence of malignant lesions (sensitivity = 1, specificity = 0.9).

**Conclusion:**

Thermography can serve as an effective non‐invasive diagnostic modality for detecting suspicious oral lesions by leveraging temperature differences. The designed device facilitates early detection of these lesions based on thermal variations, offering a promising tool for timely and accurate diagnosis.

**Trail Registration:** IRCT20181130041806N1

## 1 Introduction

The World Health Organization (WHO) designates oral cancer as a top priority in its initiatives for non‐communicable diseases and universal health coverage. According to the GLOBOCAN 2022 project, based on updated estimates from the International Agency for Research on Cancer (IARC) of the WHO, the estimated incidence of lip and oral cancer is 389 485, and its estimated mortality is 188 230 [1]. According to the Iranian National Cancer Registry (INPCR) in 2016, Iran recorded 124 833 new cancer cases, with 52.90% reported in men and 47.10% in women [2]. The age‐standardized rate (ASR) for oral cavity cancer was 1.96 and 1.36 per 100 000 for Iranian men and women, respectively [3]. Oral squamous cell carcinoma (OSCC) accounts for over 90% of oral cancers [4, 5, 6, 7]. The highest risk sites include the lateral border of the tongue and the floor of the mouth [8].

The five‐year survival rate of OSCC for cases detected at early stages is reported to be as high as 75%–94%. However, most patients are diagnosed in their late advanced stages due to limited and complex early diagnostic modalities, reducing the survival rate to 20%–40% [9, 10, 11, 12]. Identifying signs and symptoms during an initial oral screening is crucial, as the timing of these screenings significantly influences disease prognosis and enhances patient survival prospects [13].

Incisional biopsy remains the gold standard for diagnosing oral lesions. However, the accuracy of this method is highly dependent on the physician's expertise in planning and performing the biopsy, particularly in large or complex lesions, as well as the clinician's skill in processing tissue specimens. Additionally, biopsy is an invasive procedure, posing challenges for medically compromised patients [14, 15, 16, 17, 18, 19, 20, 21].

To leverage the unique properties of cancer cells, adjunct diagnostic modalities such as optical imaging systems, toluidine blue dye, oral CDX brush biopsy kits, and blood and saliva analyses have been developed. These methods facilitate the early detection of oral lesions, providing less invasive alternatives to traditional biopsy. A higher rate of metabolism and angiogenesis activity in tumoral tissues, compared to healthy tissues, results in a notable temperature difference between the two. This characteristic has been less commonly studied for the detection of oral cancer [10, 11, 22, 23, 24]. Thermographic cameras have long exploited this property for the non‐invasive detection of breast and skin cancers [22, 25, 26, 27, 28]. Digital infrared thermal imaging (DITI) has also been employed to detect oral cancer according to the metabolic activity of the tumoral tissues [10, 11, 29].

This study aimed to evaluate the sensitivity and specificity of a novel, custom‐designed, non‐invasive diagnostic modality for detecting intraoral tumoral lesions using thermal distribution measured by an IR sensor. The device was specifically designed and manufactured to determine the temperature difference between healthy and tumoral tissues to facilitate OSCC detection.

## 2 Patients and Methods

### 2.1 Study Design

This clinical trial study evaluated the accuracy of a non‐invasive diagnostic modality for detecting OSCC by measuring the temperature difference between healthy and tumoral tissues using an IR sensor. To the best of our knowledge, this is the first study to utilize an IR sensor as a rapid, non‐invasive, and non‐ionizing modality for the detection of oral cancer. The study was conducted at the Cancer Institute of the Imam Khomeini Hospital Complex, a tertiary cancer center within the Imam Khomeini Hospital Complex affiliated with Tehran University of Medical Sciences (Tehran‐Iran), from October 2016 to December 2018.

### 2.2 Study Population

Participants for this study were recruited from the Tehran region in Iran, adhering to specific inclusion and exclusion criteria. The inclusion criteria for the patient group included individuals over 10 years of age who were clinically suspected of having OSCC and presented to the Cancer Institute of Imam Khomeini Hospital. Exclusion criteria included current users of any form of tobacco, including smoking, chewing tobacco, and betel quid, as well as individuals with a history of oral lesions such as periodontitis, precancerous conditions, or recurrent oral cancer. Periodontal disease was specifically ruled out based on clinical attachment loss and radiographic bone loss evaluation. Participants with prior chemotherapy, radiotherapy affecting the head and neck region, or oral surgery were excluded due to potential alterations in tissue characteristics that could affect thermal measurements. Acute infections and systemic conditions affecting thermoregulation, including diseases of the sweat glands such as ectodermal dysplasia, were also considered exclusion criteria. Additionally, individuals presenting with fever or systemic hyperthermia at the time of examination were excluded to prevent systemic temperature fluctuations from confounding the analysis. Furthermore, vascular malformations such as hemangiomas were ruled out to ensure that localized anomalies in blood flow did not affect thermal assessment.

To further minimize potential confounders, an oral medicine specialist performed a comprehensive clinical evaluation, rigorously confirming the absence of any other oral diseases or conditions that might interfere with the thermographic analysis.

**FIGURE 1:**
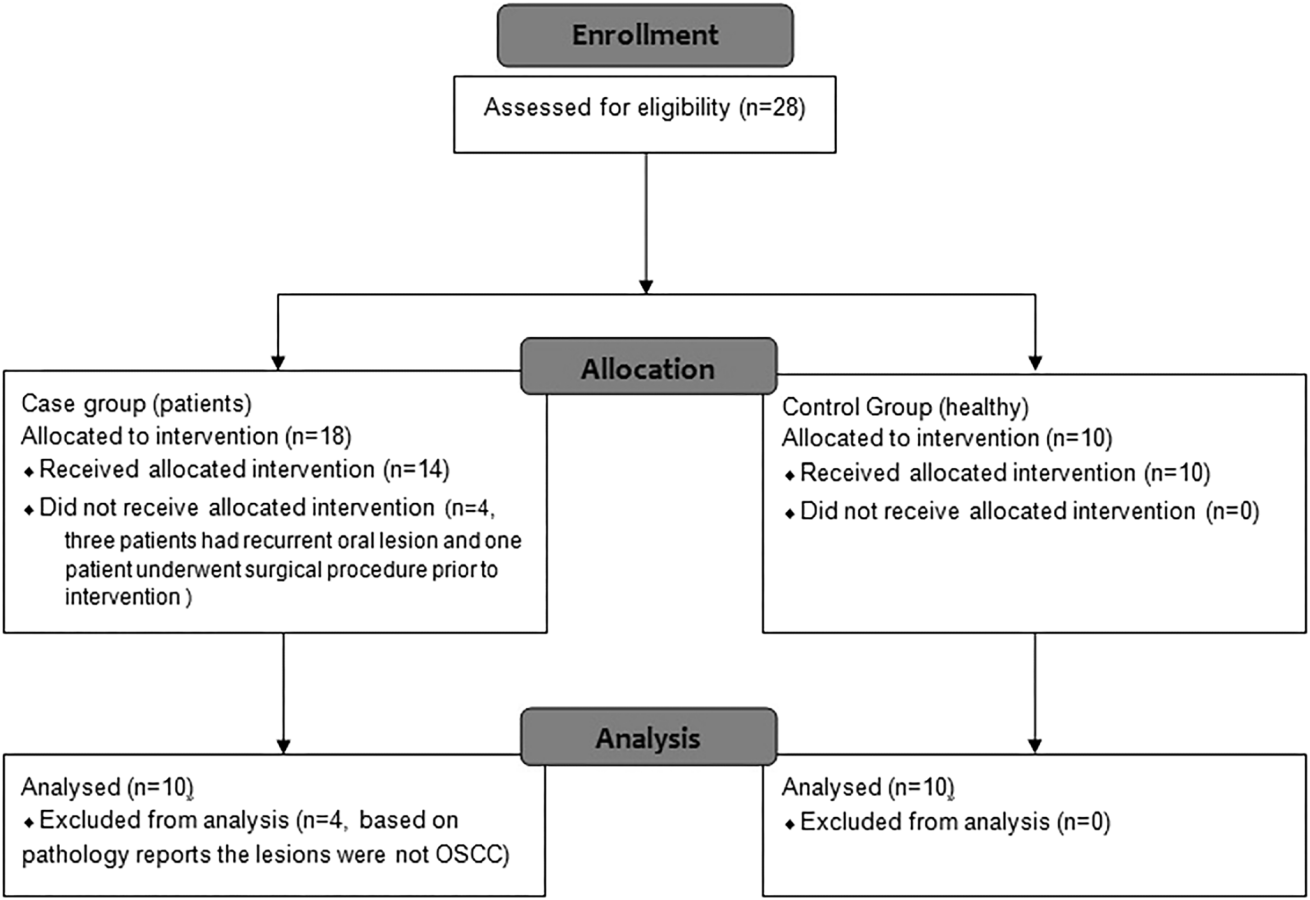
Diagram of patients included in the study.

A total of 28 participants were initially selected, including 19 males and nine females. Eight patients were excluded based on the exclusion criteria. Consequently, a total of 10 patients (six males and four females) were chosen for the patient group to diagnose their oral lesions. The control group included 10 healthy individuals (seven males and three females) with no history of oral cancer or other suspected lesions, as determined during routine clinical examination (Figure 1). Written informed consent was obtained from all participants.

The sample size for this study was determined based on the results from an initial pilot study involving 10 cases. To detect a significant temperature difference of 0.10°C between case and control groups, a minimum sample size of eight participants per group was calculated. This calculation was performed using the independent sample *t*‐test tab of PASS 11 software, with a significance level (*α*) of 0.05 and a power (*β*) of 0.1.

This study was approved by the ethics committee of the Tehran University of Medical Sciences as a clinical trial study with the registration number IR.TUMS.VCR.REC.1395.473 and the Iranian Registry of Clinical Trial number IRCT20181130041806N1.

### 2.3 Intervention

A novel non‐invasive device was designed and manufactured incorporating an infrared sensor to accurately assess the heat difference between healthy and tumoral tissues. A miniaturized medical grade infrared thermometer sensor (Melexis MLX90614) was selected for this study to meet the clinical requirements of accuracy, continuous data collection and ease of use. The sensor and the device were calibrated according to the manufacturer's instructions and cross‐validated with a commercial‐grade thermometer in a laboratory setting to verify the accuracy of the readings.

The device was designed with a small, flexible head that allows for easy maneuverability and access to all areas within the oral cavity. This is a significant advantage over currently available thermographic devices, which are not suitable for intraoral use due to their limitations in site‐specific detection. This novel device boasts high accuracy, utilizing an MLX90614 infrared sensor with a measurement resolution of 0.02°C and an accuracy of 0.3°C. It continuously displays temperature measurements on a monitor and features an alert system that triggers an alarm when the temperature exceeds the defined average oral temperature (37.7°C in men and 38.1°C in women) [30, 31]. Designed according to ergonomic dental principles and ISO 13485 regulations for medical devices, this device is user‐friendly for clinicians. Additionally, it is portable, wireless, and powered by a rechargeable battery, eliminating the need for external power sources, cameras, laptops, or specific setups. This portability is crucial for use in remote areas with limited access to power and other resources.

For standardization, participants were instructed to refrain from eating and drinking for 30 min prior to data collection. Additionally, to stabilize metabolic activity, participants were asked to sit upright in a well‐lit room with optimal temperature and humidity (24°C) for 20 min. Measurements were recorded by an expert oral medicine specialist trained specifically for this study. Data collection commenced by capturing participants' core body temperature sublingually using sterilized conventional mercury thermometers. The patients were asked to maintain a seated position with their heads slightly tilted backward. This positioning maximized the device's probing access and enhanced the examiner's field of view. The device was then activated, and the sensor was held at a close distance from the center of the lesion. According to the literature, this area tends to exhibit the highest temperature [32]. The sensor was held in place for approximately 30 s until the temperature displayed on the monitor stabilized. This process was repeated three times to minimize measurement error, and the mean of the measurements was reported as the mean temperature of the lesion. The same procedure was applied to the contra‐lateral healthy mucosa of the same patient, with the temperature recorded as the control. For infection control, the device's tip was covered with disposable covers for each patient following the infection control protocol. Ultimately, all patients underwent an incisional biopsy, the gold standard for confirming a clinical diagnosis [14, 17, 18, 19, 33]. A total of 18 patients were initially evaluated; three patients were excluded due to recurrent lesions; four were excluded because the pathology report indicated the lesion was not OSCC, and one patient was excluded due to undergoing a surgical procedure prior to the measurements. Subsequently, the temperature at corresponding sites was measured in 10 healthy controls, as previously described. The thermal difference between the tumor site and healthy mucosa in patients with histopathologically confirmed OSCC was analyzed and compared with the thermal difference between the two sides of the tongue in healthy controls. Data analysis was conducted using SPSS version 16.

### 2.4 Statistical Analysis

This study included 10 patients with oral lesions and 10 healthy controls. A *t*‐test was used to compare the temperature between the two sides in the case and control groups. The results showed no significant difference in temperature between the two sides in healthy controls (mean difference of 0.03°C, *p* = 0.914). Consequently, the temperature of the right side of the tongue in the control group was compared with the temperature of the lesion side in the patient group. Independent sample *t*‐tests were employed to compare the temperatures between the case and control groups. Pearson's correlation test was used to evaluate the relationship between the lesion temperature, the control side temperature, the temperature difference between the two sides, and the body temperature of the two groups. *p*‐values less than 0.05 were considered statistically significant.

Additionally, an ancillary analysis was conducted to determine the sensitivity and specificity of the designed device. The area under the receiver operating characteristic (ROC) curve was calculated to assess the diagnostic performance of the device.

## 3 Results

A total of 28 participants, including 19 males (68%) and 9 females (32%) with a mean age of 43 ± 26 years, were initially recruited. Among these, 18 participants were patients with oral squamous cell carcinoma (OSCC), including 12 males and six females. According to the exclusion criteria, eight patients were excluded leaving 10 patients (six males and four females) in the study. Of these, eight patients had SCC of the tongue, and two had SCC of the lip. There were no cases of SCC in other parts of the oral cavity, such as the floor of the mouth, palate, or gingiva. At the time of the study, the patients had lesions for an average duration of 5 months. Seventy percent of the patients had a history of smoking, alcohol consumption, substance abuse, or passive smoking, while 30% had no history of tobacco use or passive smoking. Additionally, 70% of the participants had a history of cancer in their first‐degree or second‐degree relatives.

**FIGURE 2:**
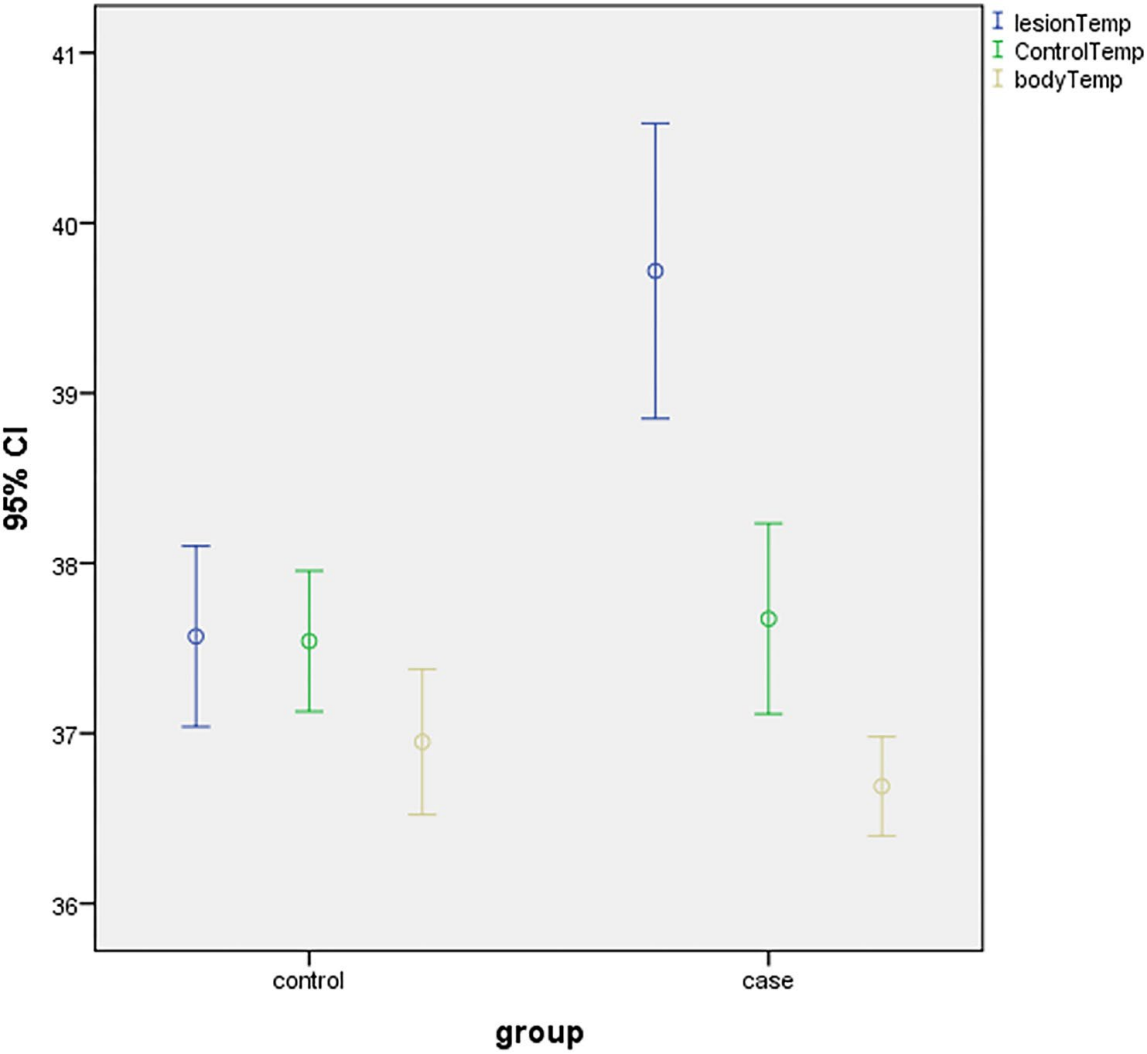
Diagram of error bar of the mean and 95% confidence interval of the temperature in the lesion and control sides and the body temperature in the case and control groups.

The control group included 10 healthy individuals, comprising seven males and three females. In this group, the temperature of the right and left sides of the tongue was measured. No significant difference was noted between the temperature of the two sides of the tongue (*p* = 0.860). However, in the test group, the mean temperature difference between the lesion and the contralateral healthy side was 2.06°C ± 0.85°C, which was statistically significant (*p* < 0.001). The temperature at the tumor site was 1.1 to 3.42°C higher than the mean temperature of the healthy mucosa (Tables 1 and 2; Figure 2).

**TABLE 1:** Mean temperature of the two sides in the case and control groups (*n* = 10).

Group			Mean	Std. deviation	Std. error mean
Control	Pair 1	Right temperature	37.5700	0.74172	0.23455
		Left temperature	37.5420	0.57823	0.18285
Case	Pair 1	Lesion temperature	39.7520	1.07058	0.33855
		Control temperature	37.6900	0.68911	0.21791

**TABLE 2:** Comparison of the mean difference in temperature of the two sides in the case and control groups.

Group			Paired differences					*t*	df	Sig. (2‐tailed)
			Mean	Std. deviation	Std. error mea*n*	95% confidence interval of the difference				
						Lower	Upper			
Control	Pair 1	Right temperature—left temperature	0.02800	0.48810	0.15435	−0.32116	0.37716	0.181	9	0.860
Case	Pair 1	Lesion temperature—control temperature	2.06200	0.85559	0.27056	1.44995	2.67405	7.621	9	0.000

**FIGURE 3:**
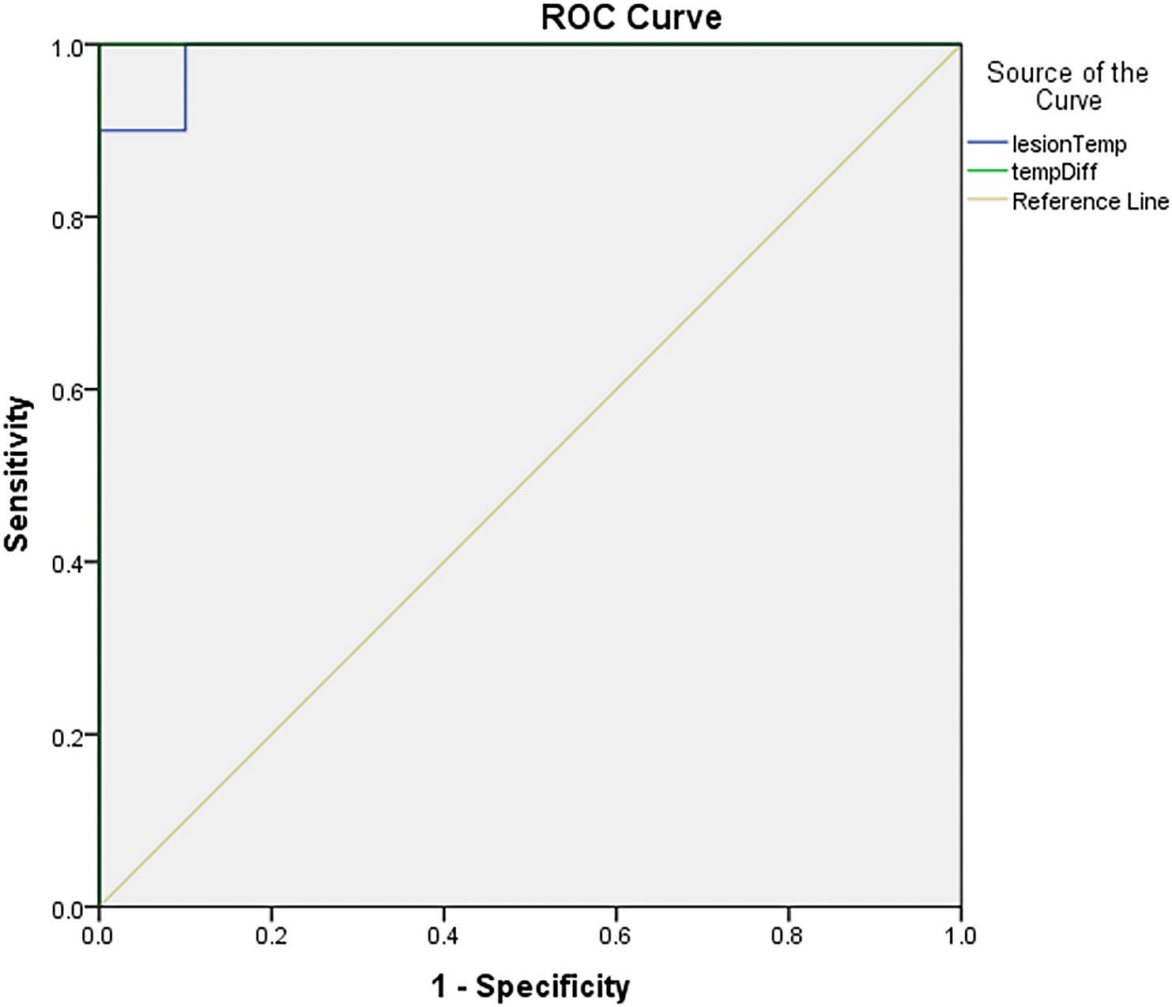
Diagram of sensitivity and specificity of the designed device according to the temperature of the lesion and thermal difference.

According to the ROC curve analysis, if the temperature difference between the two sides of the oral cavity exceeds 0.97°C, while the body temperature is within the normal range, there is a higher likelihood of a lesion being present on the side with the higher temperature. This criterion demonstrated a sensitivity and specificity of 1.0 (Table 3; Figure 3). Additionally, areas with a temperature exceeding 38.42°C may indicate the presence of an oral lesion, with a sensitivity of 1 and a specificity of 0.9. Based on the findings of this study, such lesions could potentially be identified as oral squamous cell carcinoma.

**TABLE 3:** ROC curve to assess the sensitivity and specificity of the designed device based on the temperature of the lesion and the difference in temperature.

Test result variable(s)	Positive if greater than or equal to^a^	Sensitivity	1−specificity
Lesion temperature	35.2800	1.000	1.000
	36.5600	1.000	0.900
	37.0100	1.000	0.800
	37.2900	1.000	0.600
	37.5000	1.000	0.500
	37.8100	1.000	0.400
	38.0900	1.000	0.300
	38.2500	1.000	0.200
	38.4200	1.000	0.100
	38.6000	0.900	0.100
	38.7200	0.900	0.000
	38.8000	0.800	0.000
	39.1100	0.700	0.000
	39.4300	0.600	0.000
	39.6000	0.500	0.000
	39.7500	0.400	0.000
	39.9300	0.300	0.000
	40.5300	0.200	0.000
	41.5000	0.100	0.000
	43.0000	0.000	0.000
Temperature difference	−0.9200	1.000	1.000
	0.1400	1.000	0.800
	0.2100	1.000	0.700
	0.2400	1.000	0.600
	0.2800	1.000	0.500
	0.3900	1.000	0.400
	0.5600	1.000	0.300
	0.6900	1.000	0.200
	0.7900	1.000	0.100
	0.9700	1.000	0.000
	1.1100	0.900	0.000
	1.1900	0.800	0.000
	1.3100	0.700	0.000
	1.6300	0.600	0.000
	2.0600	0.500	0.000
	2.3500	0.400	0.000
	2.5100	0.300	0.000
	2.8800	0.200	0.000
	3.3200	0.100	0.000
	4.4200	0.000	0.000

^a^ The smallest cutoff value is the minimum observed test value minus 1, and the largest cutoff value is the maximum observed test value plus 1. All the other cutoff values are the averages of two consecutive ordered observed test values.

## 4 Discussion

Oral cancer is one of the 10 most common causes of morbidity and mortality worldwide [4, 15], with approximately 188 230 deaths annually attributed to the disease [1]. It is, therefore, considered a global health dilemma. Due to the late diagnosis of oral cancer, its 5‐year survival rate remains around 50%, which is lower than that of colorectal cancer, cervical cancer, and breast cancer [15, 18]. This highlights the importance of adjunct diagnostic methods for detecting oral cancer in its early stages. Evidence shows a direct correlation between the sensitivity of thermography and tumor metabolism and its size. Cancer cells exhibit a higher metabolic rate and require an increased blood supply, resulting in a temperature difference between the tumoral site and the healthy tissue [16]. While this characteristic has been extensively utilized for the detection of breast and skin cancer, it has been less commonly applied for the detection of oral cancer [10, 11]. Since abnormal temperature patterns can be readily detected by an infrared (IR) thermometer, early diagnosis by thermography is possible and can be integrated into dental offices to identify malignancies. With advancements in IR detectors, IR thermography is increasingly being adopted as a diagnostic tool for assessing abnormal thermal patterns [22, 25, 27]. The present study utilized this technology and found that the temperature of the tumoral lesion was 1.1 to 3.42°C higher than the mean temperature of the healthy oral mucosa. These findings suggest that an IR thermometer can effectively detect lesion areas and differentiate them from healthy oral mucosa.

White et al. conducted one of the earliest studies on the potential of IR thermography for assessing normal and abnormal oral mucosa in 1986, using a Hughes Series 4000 PROBEYE thermal video system, an IR scanner, and a microprocessor. They observed that the temperature of normal mucosa was significantly lower than that of oral lesions. They demonstrated that each lesion elevated the temperature of its surrounding tissues, with the highest temperature recorded at the lesion's center. Their findings showed that superficial temperature differences may indicate inflammatory changes related to oral lesions and can be accurately measured by thermography [32].

Moustafa et al., in 2012, evaluated two groups of laboratory animals with cancer cells injected into their bodies. Their results showed that thermography could detect skin cancer, with the tumoral site exhibiting a significantly higher temperature of 0.3°C–0.5°C than the mean temperature [28]. Since tumoral cells in all body parts exhibit higher rates of metabolism and angiogenesis—and oral cancer, similar to skin cancer, occurs on the superficial layers of the human body—the temperature difference between lesions and healthy mucosa may aid in detecting oral cancerous lesions and distinguishing them from healthy tissues. Like skin cancer, oral cancer often goes undetected despite being visible and expected to be identified during routine clinical examinations. This contributes to a low 5‐year survival rate and underscores the need for multiple diagnostic modalities for effective detection [34]. The present study found that the temperature of tumoral lesions was significantly higher than that of healthy mucosa. This finding aligns with the result of Moustafa et al. [28], who detected skin cancer using thermographic measurements of tumoral tissue thermal alterations. Similar to our study, they utilized thermography to detect cancer in superficial body layers, demonstrating significant temperature between tumoral lesions and healthy tissues.

Chakraborty et al. [10, 11, 29] employed IR digital thermal imaging to detect oral cancer. They used heat distribution asymmetry on the face as a key parameter for identifying oral cancer. Their findings showed that due to the higher metabolic activity of tumoral regions, patients with oral cancer exhibited greater heat distribution asymmetry compared to healthy individuals. Their results were consistent with our findings, supporting the use of thermography for oral cancer detection based on temperature differences between tumoral and healthy tissues.

Garduño‐Ramón et al., in 2017, assessed the accuracy of thermographic cameras for detecting breast cancer. They found that thermography could serve as an adjunct tool for detecting breast cancer and angiogenesis, with a sensitivity of 0.78 and a specificity of 0.80. These findings confirmed that thermography is an effective diagnostic modality for identifying malignancies in internal organs [35].

Moradi et al., in 2007 [36], demonstrated that the sensitivity of thermographic imaging remains consistent under variable environmental conditions. Parameters such as skin radiation coefficient, skin moisture, air temperature, air humidity percentage, and thermal conductivity do not significantly affect the sensitivity of thermography. Thus, the presence of a fluid such as saliva does not cause errors in this process.

A key strength of the current study is the use of a customized probe and device capable of capturing continuous oral temperature with a measurement resolution of 0.02°C and an accuracy of 0.3°C. This device can detect subtle temperature changes in the target tissue and trigger an alarm when temperatures exceed normal levels in the scanned area.

The primary limitation of this study is its small sample size. Future research with larger sample sizes is needed to validate the reproducibility of these results using IR thermography. Additionally, studies involving patients with other malignant and premalignant oral lesions are required to establish a thermal range for these conditions and differentiate OSCC from other oral lesions.

In conclusion, this study represents a pioneering effort to use IR thermography as a non‐invasive diagnostic modality for detecting oral cancer in its early stages, with the potential to reduce the mortality rate. The findings pave the way for further research into the non‐invasive detection of malignant and premalignant oral lesions using IR thermography.

The significance of early detection of oral cancer has been emphasized by many researchers. This study introduces a method that is non‐invasive, cost‐effective, and accessible, particularly in remote areas lacking well‐trained personnel and high‐tech equipment.

To validate these findings, future research should include larger sample sizes and longitudinal studies. Nonetheless, this study offers a promising new approach for early detection of oral cancer and other oral lesions.

## Author Contributions

**Shahrzad Rahimizadeh Nahavandi:** writing – original draft, project administration, investigation, visualization, writing – review and editing. **Arghavan Tonkaboni:** writing review and editing, supervision, methodology, validation. **Soheila Manifar:** supervision, investigation, methodology, validation. **Mohammad Shirkhoda:** methodology, validation. **Amir Parham Pirhadi Rad:** conceptualization, resources, software, writing – review and editing.

## Funding

The authors have nothing to report.

## Disclosure

This is a new submission. This work has not been previously published (except as part of an academic thesis) in any language. It is not under simultaneous consideration by another journal or in the press. All authors have approved its publication.

## Conflicts of Interest

The authors declare no conflicts of interest.

## Data Availability

The data that support the findings of this study are available from the corresponding author upon reasonable request.

## References

[1] F. Bray , M. Laversanne , H. Sung , et al., “Global Cancer Statistics 2022: GLOBOCAN Estimates of Incidence and Mortality Worldwide for 36 Cancers in 185 Countries,” CA: A Cancer Journal for Clinicians 74, no. 3 (2024): 229–263. 38572751 10.3322/caac.21834

[2] F. Hadavandsiri , L. Allahqoli , Y. Rahimi , H. Salehiniya , E. Ghazanfari Savadkoohi , and M. E. Akbari , “Cancer Incidence in Iran in 2016: A Study Based on the Iranian National Cancer Registry,” Cancer Reports (Hoboken) 7, no. 2 (2024): e1967. 10.1002/cnr2.1967 PMC10850001 38148563

[3] M. Jokar , N. Namavari , S. A. Moshiri , H. K. Jahromi , and V. Rahmanian , “The Incidence of Oral Cavity Cancer in Iran: A Systematic Review and meta‐Analysis,” Cancer Reports (Hoboken) 6, no. 6 (2023): e1836. 10.1002/cnr2.1836 PMC10242657 37191384

[4] E. F. de Morais , R. P. Mafra , A. K. Gonzaga , et al., “Prognostic Factors of Oral Squamous Cell Carcinoma in Young Patients: A Systematic Review,” Journal of Oral and Maxillofacial Surgery 75, no. 7 (2017): 1555–1566. 28061358 10.1016/j.joms.2016.12.017

[5] B. W. Neville , D. D. Damm , C. M. Allen , and A. C. Chi , Oral and Maxillofacial Pathology‐E‐Book (Elsevier Health Sciences, 2023).

[6] S. Irani , “Pre‐Cancerous Lesions in the Oral and Maxillofacial Region: A Literature Review With Special Focus on Etiopathogenesis,” Iranian Journal of Pathology 11, no. 4 (2016): 303–322. 28855922 PMC5563928

[7] R. P. Langlais , C. S. Miller , and J. S. Gehrig , Color Atlas of Common Oral Diseases, 5th ed. (Wolters Kluwer (Jones & Bartlett Learning), 2016).

[8] M. Gormley , E. Gray , C. Richards , et al., “An Update on Oral Cavity Cancer: Epidemiological Trends, Prevention Strategies and Novel Approaches in Diagnosis and Prognosis,” Community Dental Health 39, no. 3 (2022): 197–205. 35852216 10.1922/CDH_00032Gormley09

[9] J. M. Hirsch , R. Sandy , B. Hasséus , and J. Lindblad , “A Paradigm Shift in the Prevention and Diagnosis of Oral Squamous Cell Carcinoma,” Journal of Oral Pathology & Medicine 52, no. 9 (2023): 826–833. 37710407 10.1111/jop.13484

[10] M. Chakraborty , S. Mukhopadhyay , A. Dasgupta , et al., “A New Paradigm of Oral Cancer Detection Using Digital Infrared Thermal Imaging,” in Medical Imaging 2016: Computer Aided Diagnosis, ed. G. D. Tourassi and S. G. Armato , vol. 9785 (International Society for Optics and Photonics, 2016).

[11] M. Chakraborty , S. Mukhopadhyay , A. Dasgupta , et al., “A New Approach of Oral Cancer Detection Using Bilateral Texture Features in Digital Infrared Thermal Images,” in 2016 IEEE 38th Annual International Conference of the Engineering in Medicine and Biology Society (EMBC) (IEEE, 2016). 10.1109/EMBC.2016.7590964 28268582

[12] M. Rutkowska , S. Hnitecka , M. Nahajowski , M. Dominiak , and H. Gerber , “Oral Cancer: The First Symptoms and Reasons for Delaying Correct Diagnosis and Appropriate Treatment,” Advances in Clinical and Experimental Medicine 29, no. 6 (2020): 735–743. 32598579 10.17219/acem/116753

[13] D. Chakraborty , C. Natarajan , and A. Mukherjee , “Advances in Oral Cancer Detection,” Advances in Clinical Chemistry 91 (2019): 181–200. 31331489 10.1016/bs.acc.2019.03.006

[14] E. Omar , “Current Concepts and Future of Noninvasive Procedures for Diagnosing Oral Squamous Cell Carcinoma‐a Systematic Review,” Head & Face Medicine 11, no. 1 (2015): 6. 25889859 10.1186/s13005-015-0063-z PMC4396078

[15] M. Rad , “Oral Cancer,” Journal of Kerman University of Medical Sciences 15, no. 4 (2008): 363–370.

[16] J. Ferlay , I. Soerjomataram , R. Dikshit , et al., “Cancer Incidence and Mortality Worldwide: Sources, Methods and Major Patterns in GLOBOCAN 2012,” International Journal of Cancer 136, no. 5 (2015): E359–E386. 25220842 10.1002/ijc.29210

[17] M. Glick , M. S. Greenberg , P. B. Lockhart , and S. J. Challacombe , eds., Burket's Oral Medicine, 13th ed. (Wiley‐Blackwell, 2021).

[18] D. V. Messadi , “Diagnostic Aids for Detection of Oral Precancerous Conditions,” International Journal of Oral Science 5, no. 2 (2013): 59–65. 23743617 10.1038/ijos.2013.24 PMC3707069

[19] Z. Delavarian , N. Mohtasham , P. Mosannen‐Mozafari , A. Pakfetrat , M. T. Shakeri , and R. Ghafoorian‐Maddah , “Evaluation of the Diagnostic Value of a Modified Liquid‐Based Cytology Using OralCDx Brush in Early Detection of Oral Potentially Malignant Lesions and Oral Cancer,” Medicina Oral, Patología Oral y Cirugía Bucal 15, no. 5 (2010): e671–e676. 20383114 10.4317/medoral.15.e671

[20] M. W. Lingen , J. R. Kalmar , T. Karrison , and P. M. Speight , “Critical Evaluation of Diagnostic Aids for the Detection of Oral Cancer,” Oral Oncology 44, no. 1 (2008): 10–22. 17825602 10.1016/j.oraloncology.2007.06.011 PMC2424250

[21] L. L. Patton , J. B. Epstein , and A. R. Kerr , “Adjunctive Techniques for Oral Cancer Examination and Lesion Diagnosis: A Systematic Review of the Literature,” Journal of the American Dental Association (1939) 139, no. 7 (2008): 896–905. 18594075 10.14219/jada.archive.2008.0276

[22] R. Usamentiaga , P. Venegas , J. Guerediaga , L. Vega , J. Molleda , and F. Bulnes , “Infrared Thermography for Temperature Measurement and Non‐Destructive Testing,” Sensors 14, no. 7 (2014): 12305–12348. 25014096 10.3390/s140712305 PMC4168422

[23] M. Anbar , “Clinical Thermal Imaging Today,” IEEE Engineering in Medicine and Biology Magazine 17, no. 4 (1998): 25–33. 9672807 10.1109/51.687960

[24] B. F. Jones , “A Reappraisal of the Use of Infrared Thermal Image Analysis in Medicine,” IEEE Transactions on Medical Imaging 17, no. 6 (1998): 1019–1027. 10048859 10.1109/42.746635

[25] N. Dey , A. S. Ashour , and A. S. Althoupety , Thermal Imaging in Medical Science Recent Advances in Applied Thermal Imaging for Industrial Applications (IGI Global, 2017), 87–117.

[26] A. M. N. Moustafa , H. H. Muhammed , and M. Hassan , “Skin Cancer Detection Using Temperature Variation Analysis,” Engineering 5, no. 10 (2013): 18.

[27] B. Lahiri , B. B. Lahiri , S. Bagavathiappan , T. Jayakumar , and J. Philip , “Medical Applications of Infrared Thermography: A Review,” Infrared Physics & Technology 55, no. 4 (2012): 221–235. 32288544 10.1016/j.infrared.2012.03.007 PMC7110787

[28] A. M. N. Moustafa , Skin Cancer Detection by Temperature Variation Analysis (M.S. thesis, School of Technology and Health, Royal Institute of Technology, 2012).

[29] M. Chakraborty , R. D. Gupta , S. Mukhopadhyay , N. Anjum , S. Patsa , and J. G. Ray , “An Introductory Analysis of Digital Infrared Thermal Imaging Guided Oral Cancer Detection Using Multiresolution Rotation Invariant Texture Features,” in Medical Imaging 2017 Computer Aided Diagnosis, ed. S. G. Armatom III and N. A. Petrick , vol. 10134 (International Society for Optics and Photonics, 2017).

[30] G. Kelly , “Body Temperature Variability (Part 1): A Review of the History of Body Temperature and Its Variability due to Site Selection, Biological Rhythms, Fitness, and Aging,” Alternative Medicine Review 11, no. 4 (2006): 278–293. 17176167

[31] M. Sund‐Levander , C. Forsberg , and L. K. Wahren , “Normal Oral, Rectal, Tympanic and Axillary Body Temperature in Adult Men and Women: A Systematic Literature Review,” Scandinavian Journal of Caring Sciences 16, no. 2 (2002): 122–128. 12000664 10.1046/j.1471-6712.2002.00069.x

[32] B. A. White , P. B. Lockhart , S. F. Connolly , and S. T. Sonis , “The Use of Infrared Thermography in the Evaluation of Oral Lesions,” International Journal of Tissue Reactions 9, no. 2 (1987): 105–114. 3610508

[33] M. W. Lingen , M. P. Tampi , O. Urquhart , et al., “Adjuncts for the Evaluation of Potentially Malignant Disorders in the Oral Cavity: Diagnostic Test Accuracy Systematic Review and meta‐Analysis‐a Report of the American Dental Association,” Journal of the American Dental Association (1939) 148, no. 11 (2017): 797–813.e52. 29080605 10.1016/j.adaj.2017.08.045 PMC7366378

[34] A. Thomas , S. Manchella , K. Koo , A. Tiong , A. Nastri , and D. Wiesenfeld , “The Impact of Delayed Diagnosis on the Outcomes of Oral Cancer Patients: A Retrospective Cohort Study,” International Journal of Oral and Maxillofacial Surgery 50, no. 5 (2021): 585–590. 32917484 10.1016/j.ijom.2020.08.010

[35] M. A. Garduño‐Ramón , S. G. Vega‐Mancilla , L. A. Morales‐Henández , and R. A. Osornio‐Rios , “Supportive Noninvasive Tool for the Diagnosis of Breast Cancer Using a Thermographic Camera as Sensor,” Sensors 17, no. 3 (2017): 497. 28273793 10.3390/s17030497 PMC5375783

[36] H. Moradi and A. Mahloojifar , “Study of Changes in Skin Temperature Distribution With Cancer Tissue Characteristics by Thermal Imaging Simulation,” 2007, https://www.civilica.com/Paper‐ICBME13‐ICBME13_017.html.

